# Coordination between zinc and phosphate homeostasis involves the transcription factor PHR1, the phosphate exporter PHO1, and its homologue PHO1;H3 in *Arabidopsis*

**DOI:** 10.1093/jxb/ert444

**Published:** 2014-01-13

**Authors:** Ghazanfar Abbas Khan, Samir Bouraine, Stefanie Wege, Yuanyuan Li, Matthieu de Carbonnel, Pierre Berthomieu, Yves Poirier, Hatem Rouached

**Affiliations:** ^1^Département de Biologie Moléculaire Végétale, Biophore, Université de Lausanne, CH-1015 Lausanne, Switzerland; ^2^Biochimie et Physiologie Moléculaire des Plantes, Institut National de la Recherche Agronomique, Centre National de la Recherche Scientifique, Université Montpellier 2, Montpellier SupAgro. Bat 7, 2 place Viala, 34060 Montpellier cedex 2, France

**Keywords:** Homeostasis, interaction, phosphate, signalling, transport, zinc.

## Abstract

Interactions between zinc (Zn) and phosphate (Pi) nutrition in plants have long been recognized, but little information is available on their molecular bases and biological significance. This work aimed at examining the effects of Zn deficiency on Pi accumulation in *Arabidopsis thaliana* and uncovering genes involved in the Zn–Pi synergy. Wild-type plants as well as mutants affected in Pi signalling and transport genes, namely the transcription factor *PHR1*, the E2-conjugase *PHO2*, and the Pi exporter *PHO1*, were examined. Zn deficiency caused an increase in shoot Pi content in the wild type as well as in the *pho2* mutant, but not in the *phr1* or *pho1* mutants. This indicated that *PHR1* and *PHO1* participate in the coregulation of Zn and Pi homeostasis. Zn deprivation had a very limited effect on transcript levels of Pi-starvation-responsive genes such as *AT4*, *IPS1*, and *microRNA399*, or on of members of the high-affinity Pi transporter family *PHT1*. Interestingly, one of the *PHO1* homologues, *PHO1;H3*, was upregulated in response to Zn deficiency. The expression pattern of PHO1 and PHO1;H3 were similar, both being expressed in cells of the root vascular cylinder and both localized to the Golgi when expressed transiently in tobacco cells. When grown in Zn-free medium, *pho1;h3* mutant plants displayed higher Pi contents in the shoots than wild-type plants. This was, however, not observed in a *pho1 pho1;h3* double mutant, suggesting that PHO1;H3 restricts root-to-shoot Pi transfer requiring PHO1 function for Pi homeostasis in response to Zn deficiency.

## Summary

Phosphate overaccumulates in shoots in response to Zn deprivation. Results shown in this article suggest key roles of PHR1 and PHO1 and a counteractive function of PHO1;H3 in controlling root-to-shoot phosphate translocation in Arabidopsis.

## Introduction

Plants require phosphorus (P) and zinc (Zn) to ensure various basic biological functions and complete their life cycle (Poirier and Bucher, 2002; Sinclair and Krämer, 2012). Plants have evolved tightly controlled mechanisms to maintain Zn and inorganic phosphate (Pi) levels in cells. Lines of evidence suggest that mechanisms for homeostasis of these two elements are interconnected and that deficiency or excess in one element impact the nutritional status of the other (Safaya and Gupta, 1979; Loneragan *et al.*, 1982; Cakmak and Marschner, 1986; Singh *et al.*, 1988; Webb and Loneragan, 1988, 1990; Loneragan and Webb, 1993; Norvell and Welch, 1993; Huang *et al.*, 2000; Zhu *et al.*, 2001).

Zn concentration in wheat and maize plants has been reported to decrease with the application of P, while Pi concentration decreases with the application of Zn (Robson and Pitman, 1983; Verma and Minhas, 1987). In barley, Zn deprivation results in overaccumulation of Pi in shoots (Huang *et al.*, 2000). Of note, the increase in Pi concentration in Zn-deficient plants can reach levels leading to Pi toxicity symptoms if high concentrations of external Pi are supplied (Loneragan *et al.*, 1982). Overaccumulation of Pi in response to Zn deficiency might partially be the result of a specific induction of the expression and activity of Pi transporters (Huang *et al.*, 2000). Taken together, these data indicate that Zn-deficient plants appear to have lost the capacity to downregulate the expression of high-affinity Pi transporters in roots despite the presence of an adequate Pi supply (Huang *et al.*, 2000). However, in *Arabidopsis*, a recent report by Jain *et al.* (2013) showed that Zn starvation causes a repression and induction of the expression of *PHT1;1* in roots and shoots, respectively.

The uptake and distribution of Pi in plants involves multiple Pi transport systems. The Pi is acquired by the root system through specific transport proteins that include the high-affinity H^+^-Pi cotransporters (PHT) (Nussaume *et al.*, 2011). In *Arabidopsis*, the *PHT1* gene family contains nine members, *PHT1;1–9*. The expression of these genes is finely regulated at the transcriptional level and depends normally on the internal P status of the plant (Muchhal and Raghothama, 1999; Smith *et al.*, 2000). Once within the root symplast, Pi can be distributed to various organelles, including the vacuoles. Alternatively, Pi can undergo symplastic transport towards the vascular cylinder for subsequent transfer to the shoot. The PHO1 protein is an important component for the root-to-shoot Pi transfer in plants, being expressed in the root vascular cylinder and mediating Pi export to the apoplast (Hamburger *et al.*, 2002). The contribution of PHO1 to the root-to-shoot Pi transport was revealed by the fact that only 3–10% of the wild-type level of Pi is translocated to shoots in the *pho1* mutant (Poirier *et al.*, 1991; Delhaize and Randall, 1995). The *Arabidopsis* genome possesses 10 homologues of *PHO1*, namely *PHO1;H1–10* (Wang *et al.*, 2004). Remarkably, only the most closely related homologue, *PHO1;H1*, could rescue the phenotype of the *pho1* mutant when expressed under the control of the *PHO1* promoter, indicating that most homologues have biological functions other than Pi transfer into the vascular cylinder (Stefanovic *et al.*, 2007). This conclusion is also supported by the wide expression pattern of *PHO1* homologues (Wang *et al.*, 2004), their differential regulation by phytohormones (Ribot *et al.*, 2008*a*, *b*), their role in development of various plant organs and their response to environmental factors, such as wounding or blue light (Kang and Ni, 2006; Ribot *et al.*, 2008b; Zhou and Ni, 2009; Zhou *et al.*, 2009). Members of the *PHO1* gene family thus appear to play important roles in different aspects of biology of plants beyond Pi export to the vascular system.

Efforts in dissecting the molecular mechanisms involved in Pi-deficiency responses in plants have considerably increased the understanding of how Pi homeostasis is regulated in plants and have led to several proposed regulatory pathways (Rouached *et al.*, 2010). In recent years, molecular mechanisms implicating regulatory microRNAs have been uncovered as a strategy to maintain Pi homeostasis in plants (Fujii *et al.*, 2005; Bari *et al.*, 2006; Chiou *et al.*, 2006; Hsieh *et al.*, 2009; Pant *et al.*, 2009). In *Arabidopsis*, a limited number of microRNA molecules have been shown to be specifically and strongly induced by Pi limitation. Particularly, microRNA399 (miR399) is characterized as a component of the shoot-to-root Pi-starvation signalling cascade (Doerner, 2008; Lin *et al.*, 2008). The mature miR399 can move from shoot to root via the phloem, where it targets the transcript of the E2-conjugase *PHO2*, leading to expression of Pi transporters (Lin *et al.*, 2008; Pant *et al.*, 2009). The increase in miR399 accumulation upon Pi deprivation is strongly suppressed in the *phr1* mutant that lacks a MYB transcription factor (Bari *et al.*, 2006). This defined a Pi-signalling cascade, in which miR399 and *PHO2* operate downstream of *PHR1* (Franco-Zorrilla *et al.*, 2004; Aung *et al.*, 2006; Bari *et al.*, 2006; Chiou *et al.*, 2006; Doerner, 2008; Lin *et al.*, 2008; Pant *et al.*, 2009). Loss-of-function mutation of PHR1 affects the expression of numerous Pi-starvation-induced genes, including the noncoding RNAs *AT4* and *IPS1* (Martín *et al.*, 2000; Shin *et al.*, 2006; Bustos *et al.*, 2010). Furthermore, PHR1 appears to be involved in the coordination of Pi homeostasis and iron in *Arabidopsis* through the regulation of the expression of the iron-storage protein FERRITIN 1 (Bournier *et al.*, 2013). Very recently, another player in the cross talk between low-Pi signalling and iron-deficiency responses in *Arabidopsis* was identified, namely the copper transport protein COPT2 (Perea-García *et al.*, 2013).

Zn is an essential micronutrient for plant growth. Zn deficiency manifests itself at physiological and molecular levels (Sinclair and Krämer, 2012). Zn serves as a highly effective cofactor for more than 300 enzymes, including the structural Zn-finger domains that mediate DNA binding of transcription factors, as well as protein–protein interactions (Sinclair and Krämer, 2012). Zn deficiency affects the content levels of essential micronutrients and macronutrients (Jain *et al.*, 2013). The transport of Zn within plant starts with its acquisition at the root periphery, followed by its loading into xylem. These transport steps rely on a diversity of gene families encoding cation transporters that play pivotal roles in Zn transport, including heavy metal ATPases (Baxter *et al.*, 2003; Hussain *et al.*, 2004), ZIPs (Grotz *et al.*, 1998; Guerinot, 2000; Palmer and Guerinot, 2009), CDFs (Mäser *et al.*, 2001) or cation antiporters (Hall and Williams, 2003). Tight regulation of intracellular Zn concentrations is a prerequisite for maintaining a functional cellular metabolism, which likely involves finely tuned regulation of these Zn transporters. A transcriptomic study revealed that Zn deficiency affects the expression of a large array of genes, in addition to those known to be involved in metal homeostasis (Van de Mortel *et al.*, 2006). Numerous observations reported the involvement of the low-molecular-weight chelator nicotianamine in the regulation of Zn homeostasis in plants (Weber *et al.*, 2004; Curie *et al.*, 2009). So far, however, it remains unclear by which mechanisms plants sense and signal Zn deficiency and how this signal affects downstream genes acting in different pathways.

Despite the accumulation of physiological and molecular evidence for the existence of an interconnection between the homeostasis of Pi and Zn in plants, the key players acting in this coordination remain unidentified. The primary aim of the present study is to broaden the understanding of the Pi–Zn signalling crosstalk in plant. The effect of Zn deficiency on Pi homeostasis has been analysed in *Arabidopsis* comparing wild-type plants with selected mutants affected in Pi sensing, signalling, and transport. Functional and gene expression analyses helped to identify important genes acting in the interconnection between Zn and Pi homeostasis in *Arabidopsis*, namely the MYB transcription factor *PHR1*, the Pi exporter *PHO1*, and its homologue *PHO1;H3*.

## Materials and methods

### Plant materials and growth conditions

The *Arabidopsis thaliana* mutants used in all experiments were in the Columbia genetic background. The previously described *phr1* mutant (Rubio *et al.*, 2001) was provided by Javier Paz-Ares (CSIC, Madrid). A 1-kbp DNA fragment (before the ATG codon) of the *PHO1;H3* promoter was fused to a β-glucuronidase (GUS) reporter gene (*pAtPHO1;H3*::GUS) and introduced into *Arabidopsis* ecotype Col-0 as previously described (Wang *et al.*, 2004). The *pho1* and *pho2* mutants were originally described by Poirier *et al.* (1991) and Delhaize and Randall (1995), respectively. Two *pho1;h3* T-DNA insertion mutant lines (SAIL_207_D02 and SALK_038711) were obtained from the European *Arabidopsis* Stock Centre (www.arabidopsis.info) (Alonso *et al.*, 2003). The homozygosity of the *pho1;h3* mutation was confirmed by PCR using the following forward and reverse primers: 5′-ATGAAGTTCGGAAAAGAGTTCTCGTC-3′ and 5′-TGGCCTTCCATTTCCAAGAGATT-3′ for SAIL_207_D02 and 5′-ATGGAGCGTGTTGAAGCAACATT-3′ and 5′-CTA GTTATCGTCATCTTCATCGTA-3′ for SALK_038711. Plants were germinated and grown in a vertical position on agar-solidified media (A1296, Sigma). The complete nutrient medium contained 0.5mM KNO_3_, 1mM MgSO_4_, 1mM KH_2_PO_4_, 0.25mM Ca(NO_3_)_2_, l00 μM NaFeEDTA, 30 μM H_3_BO_3_, l0 μM MnCl_2_, l μM CuCl_2_, 15 μM ZnSO_4_, 0.1 μM (NH_4_)_6_Mo_7_O_24_, and 50 μM KCl. Pi-deficient medium was made by replacing 1mM KH_2_PO_4_ by 1mM KCl. Zn-free medium was made by removing the only source of Zn (ZnSO_4_). Seeds were sown on the plates and stratified at 4 °C in the dark for 3 d. Plates were then transferred in a growth chamber for 20 d, day 1 of growth being defined as the first day of exposure of stratified seeds to light. Plants were grown under long-day conditions (16/8h light/dark cycle, 250 μmol·m^–2^·s^–1^, and 24/20 °C).

### Phosphate measurements

Pi measurements were performed as described by Stefanovic *et al.* (2007). Briefly, weighed fresh shoots and roots were ground separately into powder in liquid nitrogen. Ion extractions were performed in water by incubation for 30min at 70 °C. The quantification of Pi was completed by the molybdate assay according to Ames (1966).

### Real-time quantitative reverse-transcription PCR

For expression analysis, Plant RNeasy extraction kit (Qiagen) and RQ1 RNAse-free DNAse (Promega) were used to prepare total RNA free of residual genomic DNA from 100mg frozen tissue, with shoot and root being analysed separately. Total RNA was quantified with a NanoDrop spectrophotometer (Thermo Scientific). cDNA was synthesized from 2 μg total RNA using an oligo(dT) primer and M-MLV reverse transcriptase (Promega). Real-time quantitative reverse-transcription PCR (qPCR) was performed with a LightCycler 480 Real-Time PCR System using SYBR green dye technology (Roche). PCR reactions (10 μl) containing 500nM each forward and reverse primers, 5 μl SYBR Green I Master, and 3 μl of a 1:3 cDNA dilution. Reactions were performed in LightCycler 480 Multiwell Plates 384 (Roche) covered with optical film (Sarstedt). A list of primers, efficiency of amplification, and specificity of the amplified PCR products have been described by Rouached *et al.* (2011b). The following thermal profile was applied: 95 °C for 15min and 45 cycles of 95 °C for 15 s, 58 °C for 30 s,and 72 °C for 30 s. Data were analysed using the Roche LC480 software. For each gene, the relative amount of calculated mRNA was normalized to the level of the control gene ubiquitin10 mRNA (*UBQ10*: At4g05320) and expressed as relative values against wild-type plants grown in complete (+Pi and + Zn) medium. Quantification of the relative transcript levels was performed using the comparative CT method (Livak and Schmittgen, 2001; Rouached *et al.*, 2008).

### Expression analysis by promoter-GUS fusion and PHO1;H3-GFP fusion

GUS staining was performed on transgenic plants grown in agar-solidified media. Stained tissues were conserved in 70% ethanol at 4 °C after a progressive dehydration obtained from incubation in a graded series of ethanol, followed by embedding in LR White medium-grade resin or paraffin (Lagarde *et al.*, 1996). The resin sections (2 μm) were cut using a diamond knife on a Reichart ultracut microtome and paraffin sections (10 μm) were cut and then stained with the periodic acid Schiff method (Leach *et al.*, 1980). To measure GUS activity, plant samples were homogenized on ice in an enzyme extraction buffer containing 50mM NaHPO4 pH7.0, 10mM EDTA, 0.1% (v/v) Triton X-100, 0.1% (v/v) sodium lauryl sarcosine, and 10mM β-mercaptoethanol, and extracts were centrifuged at 2150 *g* and 4 °C for 10min. The GUS enzymic assay was started by mixing 100 μl GUS assay buffer (2-times diluted extraction buffer, 0.1mg/ml bovine serum albumin, 0.02% (w/v) NaN_3_, and 2mM 4-methylumberlliferyl-β-d-GlcA) with 20 μl protein extract in a 96-well microtitre plate, and was incubated at 37 °C. The amount of fluorescent product 4-methylumberlliferyl (4-MU) produced in the reaction was measured over time using a Fluoroskan-II luminescence spectrophotometer. The GUS activity was calculated as pmol 4-MU formed per μg total protein per h.

The complete *PHO1;H3* genomic coding region from 1 kbp upstream of the start codon to the penultimate codon was amplified by PCR and first cloned into the Gateway donor vector pDONR201 using BP clonase reaction and then recombined into pMDC107 (Curtis and Grossniklaus, 2003) using LR clonase (Invitrogen) to create a fusion with GFP. The resulting clone was confirmed by sequencing, introduced into *Agrobacterium tumefaciens* pGV3101, and transformed into the *pho1;h3* mutant by the floral dip method (Clough and Bent, 1998). A PHO1;H3-GFP construct was also introduced into the pMDC32 vector (Curtis and Grossniklaus, 2003) for transient expression in tobacco (*Nicotiana benthamiana*) leaves. Localization of PHO1;H3-GFP in *Arabidopsis* root and its colocalization with various marker proteins in transiently transformed tobacco epidermal cells was performed using a Zeiss LSM 700 confocal microscope with an Apochromat ×63 water immersion DIC objective with a 1.2 NA. Binary vectors with cDNA of the Golgi marker ManI-RFP (AT1G51590) (Nebenfuhr *et al.*, 1999), the plasma membrane marker CBL1-OFP (AT4G17615) (Batistic *et al.*, 2010), the *trans*-Golgi marker SYP61-RFP (AT1G28490) (Sanderfoot *et al.*, 2001), the endoplsmic reticulum marker ER-rk-mCherry (signal peptide of AtWAK2:mCherry:HDEL) (Nelson *et al.*, 2007), and the nuclearmarker histone H2B-RFP (AT5G22880) (Sridhar *et al.*, 2007) were used. Transient expression in tobacco leaves was performed as described by Arpat *et al.* (2012).

### Statistical analysis

Statistical analysis was performed through analysis of variance (ANOVA) and using the Tukey’s test to compare mean values.

## Results

**Fig. 1.**
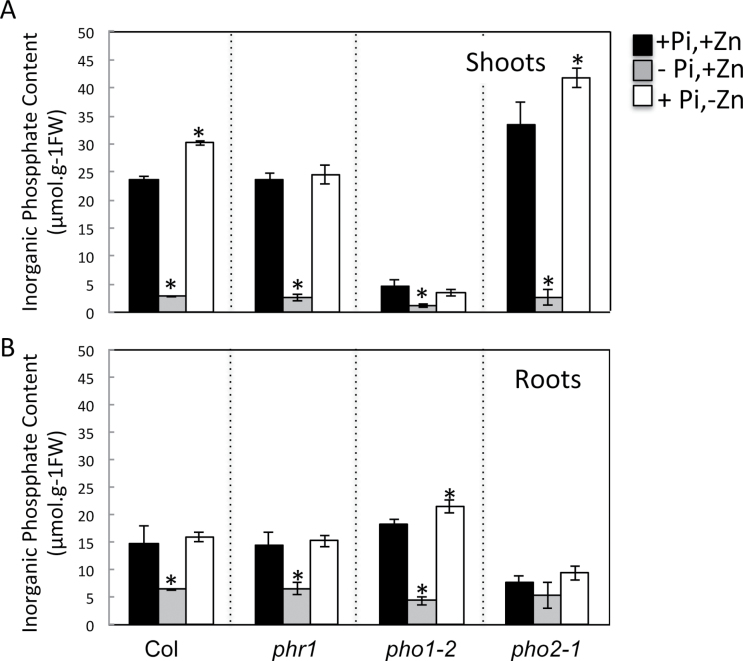
Effect of Zn availability on Pi contents in roots and shoots of *Arabidopsis*. Wild-type and *phr1*, *pho1-*2 and *pho2-1* mutant plants were grown vertically on agar-solidified media containing 1mM Pi and 15 μM Zn (+Pi,+Zn), 15 μM Zn and no Pi (–Pi,+Zn), or 1mM Pi and no Zn (+Pi,–Zn). Pi concentrations were quantified in shoots (A) and roots (B) of 20-d-old plants. Individual measurements were obtained from the analysis of shoots or roots collected from a pool of at least seven plants. Error bars correspond to standard deviation from three biological replicates. Asterisks indicate statistically significant differences compared to the +Pi,+Zn treatment within each genotype (*P* < 0.05).

Zn deficiency increases the Pi content in the shoots of *Arabidopsis* wild-type plants and *pho2* mutant plants but not in *phr1* and *pho1* mutant plants. In order to determine the effect of Zn deficiency on Pi distribution in *Arabidopsis* tissues, plants were grown in media with either low Zn (0 μM Zn) or low Pi (10 μM Pi) and compared to those grown on complete medium (1mM Pi and 15 μM Zn). Mutants affected in interorgan Pi distribution or Pi signalling were included in this experiment, namely *pho1*, *pho2*, and *phr1*. The *pho1* and *pho2* mutants are characterized by accumulation of low Pi and high Pi in shoots, respectively (Poirier *et al.*, 1991; Delhaize and Randall, 1995). The *phr1* mutant is affected in the responses to Pi deficiency, including alteration of the expression of several Pi-starvation-induced genes (Rubio *et al.*, 2001). Shoots and roots of 20-d-old plants were collected separately and Pi contents were determined (Fig. 1). As expected, Pi starvation led to a decrease in Pi content in shoots and roots. Interestingly the Pi concentration increased by 25% in shoots of wild-type and *pho2* plants grown in Zn-deficient medium compared to Zn-sufficient medium. No change in Pi concentration was however observed in the shoots of *phr1* and *pho1* mutants grown on Zn-sufficient or Zn-deficient media (Fig. 1). In roots, Zn deprivation had no impact on Pi accumulation, except for the pho1 mutant in which a 15% increase in Pi concentration (*P* < 0.05) was observed. Overall, these results imply the existence of a process leading to the accumulation of Pi in the shoots of plants under Zn deprivation. PHO1 and PHR1 participate in this process.

**Fig. 2.**
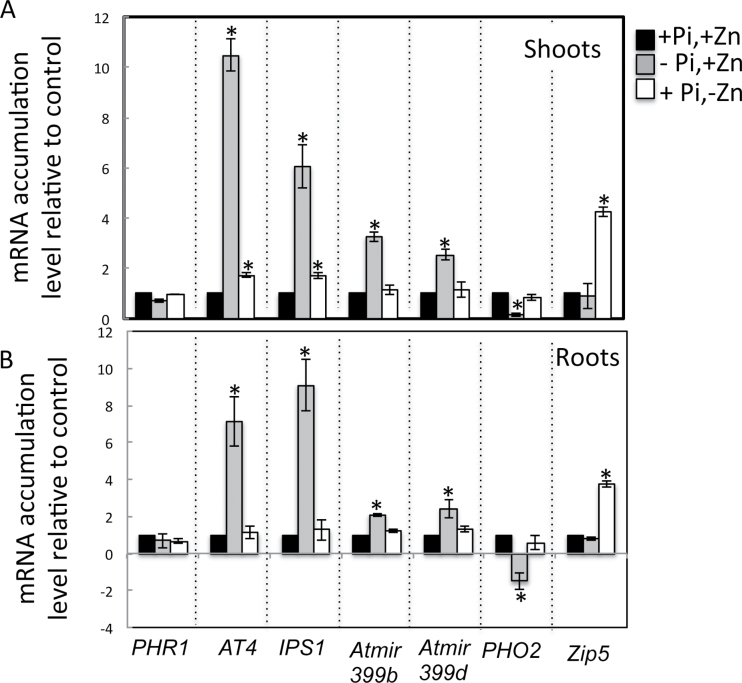
mRNA accumulation of *PHR1*, *AT4*, *IPS1*, *miR399*, *PHO2*, and *ZIP5* in response to the availability of Pi and Zn in shoots (A) and roots (B). Wild-type plants were grown vertically for 20 d on agar-solidified media containing 1mM Pi and 15 μM Zn (+Pi,+Zn), 15 μM Zn and no Pi (–Pi,+Zn), or 1mM Pi and no Zn (+Pi,–Zn). mRNA accumulation was quantified by quantitative reverse-transcription PCR. mRNA abundance of *PHR1*, *AT4*, *IPS1*, miR399b, miR399d, *PHO2*, and *ZIP5* was normalized to mRNA abundance of the *UBQ10* control gene and expressed as relative values against wild-type plants grown in +Pi,+Zn medium. Individual measurements were obtained from the analysis of shoots or roots collected from a pool of at least 10 plants. Error bars correspond to standard deviation from three biological replicates. Asterisks indicate statistically significant differences compared to the +Pi +Zn treatment for each gene (*P* <0.05).

The PHR1–miR399–PHO2 signalling pathway is not involved in the Pi response to Zn deficiency. To explore whether the PHR1–miR399–PHO2 signalling pathway is part of the Zn-deficiency response in *Arabidopsis*, this work analysed the transcript abundance of several genes involved in this pathway, namely *PHR1*, miR399b, miR399d, *PHO2*, and two Pi-starvation-responsive genes that are dependent on PHR1, namely *AT4* and *IPS1* (Martín *et al.*, 2000; Shin *et al.*, 2006). The expression of these genes was tested by qPCR for responsiveness to low versus high Zn or Pi in shoots and roots of *Arabidopsis* wild-type plants (Fig. 2). ZIP5 was also included as a positive control for testing the response to Zn deficiency (Jain *et al.*, 2013). As expected, *AT4*, *IPS1*, miR399b, and miR399d were upregulated and *PHO2* was downregulated in response to low Pi. In response to Zn deprivation, *ZIP5* was upregulated by 3–4-fold in roots and shoots, indicating that plants were indeed experiencing Zn deficiency. Under these growing conditions, there was no change in the transcript level of the Pi-starvation-response genes in either shoots or roots, except for a small but significant increase in the expression of *AT4* and *IPS1* in shoots. These results show that Zn deficiency has only a minor effect on the PHR1-dependent Pi-starvation-signalling pathway.

**Fig. 3.**
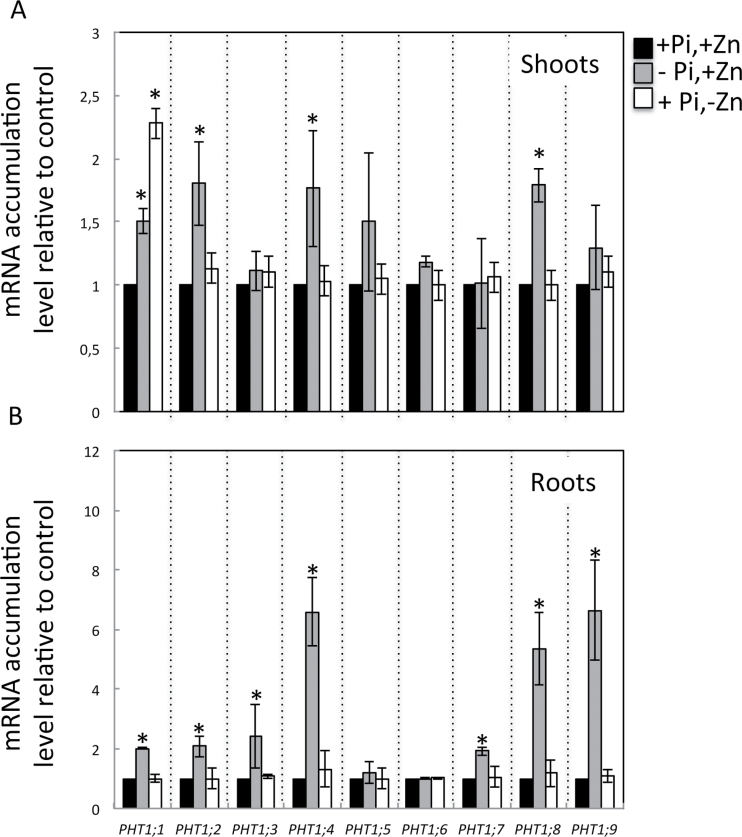
mRNA accumulation of members of the *PHT1* high-affinity phosphate transporter family in response to the availability of Pi and Zn in shoots (A) and roots (B). Wild-type plants were grown vertically on agar-solidified media containing 1mM Pi and 15 μM Zn (+Pi,+Zn), 15 μM Zn and no Pi (–Pi,+Zn), or 1mM Pi and no Zn (+Pi,–Zn). Shoots and roots of 20-d-old plants were harvested separately and mRNA accumulation was quantified by quantitative reverse-transcription PCR. mRNA abundance of the *PHT1* genes was normalized to the mRNA abundance of the *UBQ10* control gene and expressed as relative values against wild-type plants grown in +Pi,+Zn medium. Individual measurements were obtained from the analysis of shoots or roots collected from a pool of at least 12 plants. Error bars correspond to standard deviation from three biological replicates. Asterisks indicate statistically significant differences compared to the +Pi +Zn treatment for each gene (*P* <0.05).

Zn deprivation has a limited effect on the expression of high-affinity Pi transporters *PHT1* in *Arabidopsis*. In monocotyledons, Zn deficiency has been shown to cause the upregulation of a high-affinity Pi uptake transporter, leading to the overaccumulation of Pi in the shoots (Huang *et al.*, 2000). Since Zn deprivation induces Pi overaccumulation in *Arabidopsis*, this work investigated the influence of this condition on the expression of the *Arabidopsis* high-affinity Pi transporters *PHT1*. The transcript abundance of all the members of the *PHT1* gene family was determined by qPCR in shoots and roots of *Arabidopsis* wild-type plants grown in media with low and high Zn or Pi (Fig. 3). In agreement with previously published data, expression of *PHT1;1*, *PHT1;2*, *PHT1;3*, *PHT1;4*, *PHT1;7*, *PHT1;8*, and *PHT1;9* was consistently induced in roots of Pi-deficient plants. In contrast, Zn deprivation caused no significant change in expression of the *PHT1* genes either in roots or in shoots, except for *PHT1;1* which showed a 2-fold increase in transcript accumulation in shoots. These results are in agreement with the recent report by Jain *et al.* (2013) showing that Zn deficiency (15 μM) causes an upregulation of the expression of *PHT1;1* in shoots with concurrent downregulation in roots, in comparison with Zn sufficiency (75 μM). With the exception of *PHT1;1,* which was controlled at the transcription level by both Pi and Zn status of the plant (Fig. 3), the expression of the analysed *PHT1* genes appeared to be tightly regulated by the internal Pi status in *Arabidopsis* (Muchhal and Raghothama, 1999; Nussaume *et al.*, 2011).

**Fig. 4.**
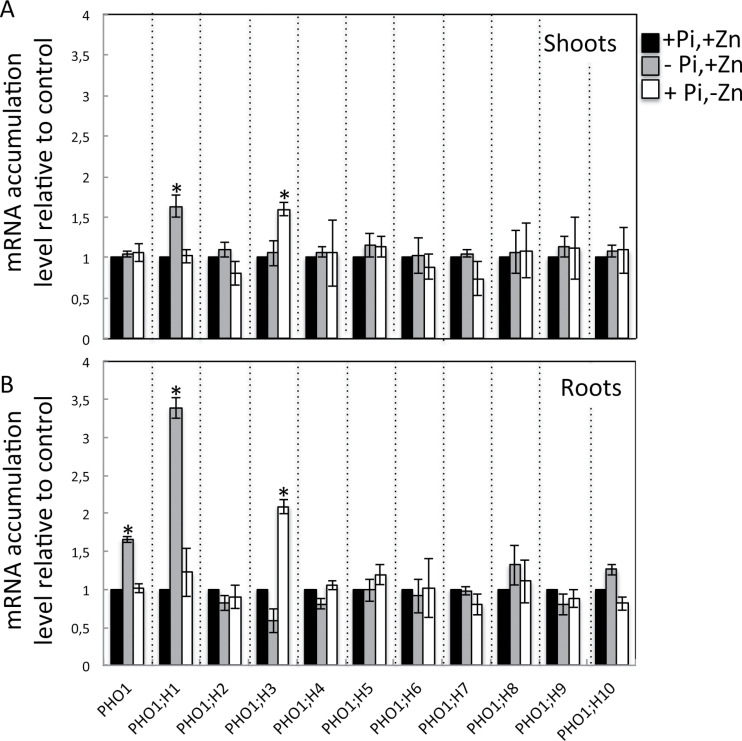
mRNA accumulation of members of the *PHO1* gene family in response to the availability of Pi and Zn in shoots (A) and roots (B). Wild-type plants were grown in a vertical position on agar-solidified media containing 1mM Pi and 15 μM Zn (+Pi,+Zn), 15 μM Zn and no Pi (–Pi,+Zn), or 1mM Pi and no Zn (+Pi,–Zn). Shoots and roots of 20-d-old plants were separately harvested and mRNA accumulation was quantified by quantitative reverse-transcription PCR. mRNA abundance was normalized to the mRNA abundance of the *UBQ10* control gene and expressed as relative values against wild-type plants grown in +Pi,+Zn medium. Individual measurements were obtained from the analysis of shoots or roots collected from a pool of at least 10 plants. Error bars correspond to standard deviation from three biological replicates. Asterisks indicate statistically significant differences compared to the +Pi +Zn treatment for each gene (*P* <0.05).

Expression of the *PHO1* homologue *PHO1;H3* is regulated by Zn deficiency. The effect of Zn deprivation on the expression of *PHO1* and its 10 homologues was explored. Transcript accumulation was analysed by qPCR in shoots and roots of wild-type plants grown in media with low and high Zn or Pi (Fig. 4). In line with previous results, *PHO1* and its closest homologue *PHO1;H1* were induced in roots and in roots and shoots, respectively, in response to Pi starvation (Stefanovic et al, 2007; Ribot *et al.*, 2008*a*). The transcript abundances of all other *PHO1* homologues were not significantly altered. In response to Zn deprivation, transcript accumulation only of *PHO1;H3* was changed, showing upregulation in both roots and shoots. Interestingly, the expression of *PHO1;H3* was not upregulated by Pi starvation. This results suggests that *PHO1;H3* plays a specific role in the regulation of Pi homeostasis under Zn deficiency.

**Fig. 5.**
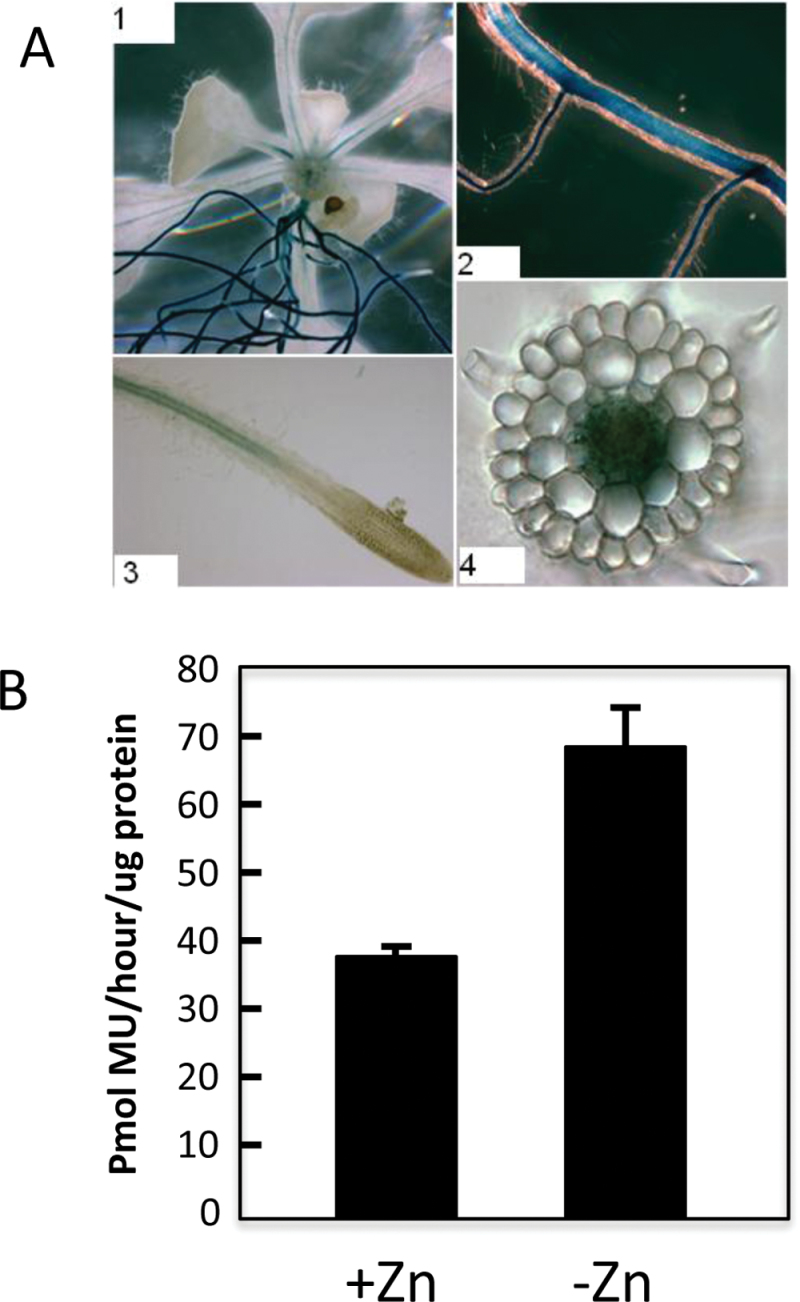
Spatial localization of *PHO1;H3* expression. (A) GUS staining of transgenic plants expressing the GUS reporter gene under the control of the *PHO1;H3* promoter grown on Zn-free agar medium; GUS expression is detectable in vascular tissues of the petiole (1) and of the primary and secondary roots (1,2), in the mature zone of the root (3), and in the root vascular cylinder (4). (B) GUS activity in plants containing the *PHO1:H3* promoter::GUS reporter; plants were grown in Zn-deficient or Zn-sufficient media for 19 d. MU, 4-methylumberlliferyl.

PHO1;H3 is expressed in root vascular cells and localized to the Golgi. In order to have a better insight into the role of *PHO1;H3* in response to Zn deficiency, this work determined its expression pattern and subcellular localization. Analysis of transgenic plants expressing the β-glucuronidase (GUS) gene under the control of the *PHO1;H3* promoter (Wang *et al.*, 2004) revealed GUS staining primarily in the vascular system of the lower section of the petiole and in the mature zones of the roots (Fig. 5A). Only weak expression in the vascular cylinder of shoots could be observed (Fig. 5A). Transversal sections of mature roots revealed the presence of GUS in cells of the stele, including the pericycle and xylem parenchyma cells (Fig. 5A). Analysis of GUS activity using 4-methylumbelliferyl-β-d-glucuronide as a substrate revealed a 1.8-fold increase in activity in plants grown under Zn-deficient media (Fig. 5B).

**Fig. 6.**
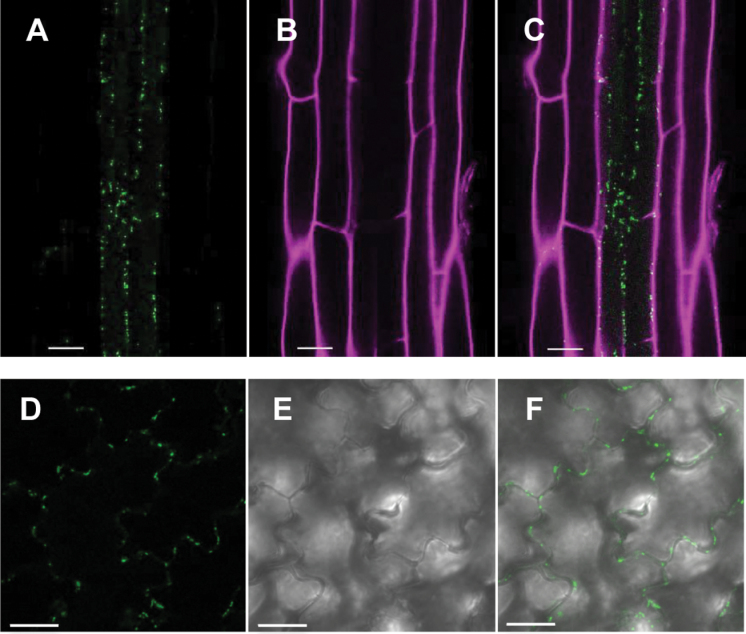
Expression pattern of PHO1;H3-GFP in *Arabidopsis*. *pho1;h3* mutant was transformed with a PHO1;H3-GFP fusion construct expressed under the *PHO1;H3* promoter and fluorescence examined in roots (A–C) and cotyledons (D–F) of 5-d-old seedlings. (A) PHO1;H3-GFP expression (green) in roots, (B) propidium iodine staining of the cell wall of root epidermal and cortical cells (magenta), and (C) overlay of A and B. (D) PHO1;H3-GFP expression (green) in epidermal cells of cotyledon, (E) transmission image, and (F) overlay of D and E. Bars = 20 μm.

**Fig. 7.**
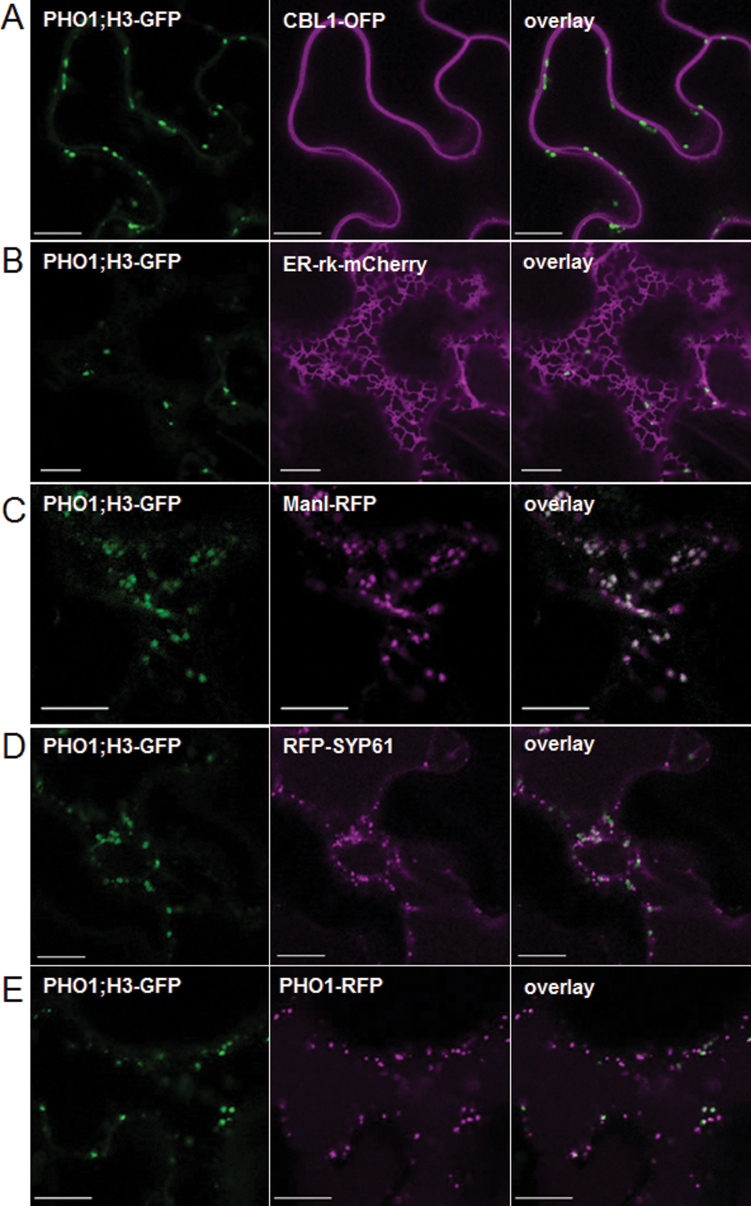
Coexpression of PHO1;H3-GFP with different subcellular markers in tobacco epidermal cells. Tobacco leaves were coinfiltrated with *Agrobacterium tumefaciens* harbouring PHO1;H3-GFP and the plasma membrane marker CBL1-OFP (A), the ER marker Er-rK-mCherry (B), the Golgi marker ManI-RFP (C), the *trans*-Golgi marker RFP-SYP61 (D), or PHO1-RFP (E). GFP signal is shown in green in the left panel while mCherry-, RFP- and OFP-signals are shown in magenta in the middle panel as indicated. Colocalization of green and magenta signals appears in white in the right panel. Bars = 10 μm.

The expression pattern of PHO1;H3 was also analysed in transgenic *pho1;h3* mutant plants expressing PHO1;H3 fused with GFP under control of its native promoter. In the root, PHO1;H3::GFP was primarily detected in cells of the vascular cylinder (Fig. 6A–C). In cotyledons, some weak expression was also observed in epidermal cells (Fig. 6D–F). At the subcellular level, PHO1;H3-GFP expression was found associated with punctate bodies. Attempts to colocalize the fluorescence pattern to particular subcellular compartments in *Arabidopsis* failed due to a combination of the relatively weak expression and difficulties in obtaining adequate resolution. As an alternative, the subcellular localization of PHO1;H3-GFP was assessed via transient expression in tobacco (*N. benthamiana*) leaves that were coinfiltrated with various subcellular markers. PHO1;H3-GFP did not colocalize with either the plasma membrane marker CBL1-OFP or the endoplasmic reticulum marker ER-rk-mCherry (Fig. 7A, B). Extensive colocalization was however observed with the Golgi marker ManI-RFP (Fig. 7C). In contrast, more limited colocalization was observed with the *trans*-Golgi (TGN) marker RFP-Syp61 (Fig. 7D). Since PHO1-GFP has previously been shown to localize to the Golgi and TGN in tobacco leaves (Arpat *et al.*, 2012), the colocalization of PHO1;H3-GFP and PHO1-RFP was analysed. The fluorescent bodies associated with PHO1;H3-GFP largely overlapped with a subset of PHO1-RFP fluorescent bodies (Fig. 7E). This pattern of overlap likely reflected the stronger association of PHO1:H3 to the Golgi while PHO1-RFP is more equally distributed to the Golgi and TGN (Arpat *et al.*, 2012). Overall, these results revealed that PHO1;H3 and PHO1 share similar and overlapping tissue and subcellular localization (Hamburger *et al.*, 2002; Arpat *et al.*, 2012). The specific upregulation of PHO1;H3 under Zn deficiency, its homology with PHO1, and the colocalization of PHO1 and PHO1;H3 suggest a potential implication of PHO1;H3 in the regulation of Pi homeostasis in response to Zn deficiency.

**Fig. 8.**
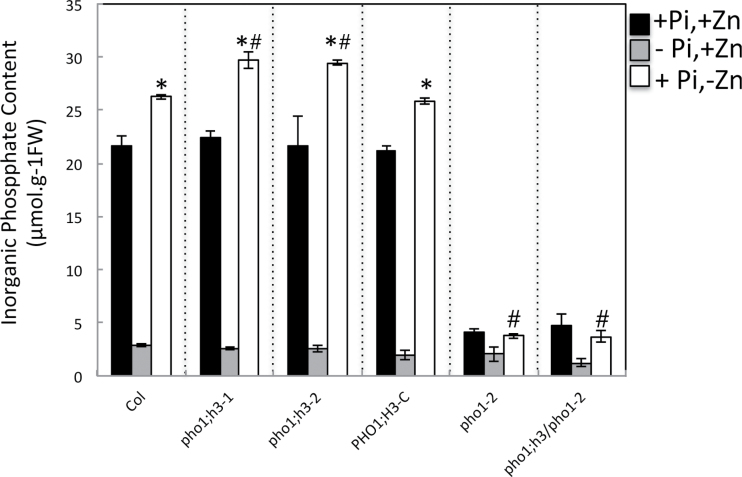
Effect of the availability of Pi and Zn on Pi content in shoots of wild-type and mutant plants. Wild-type, *pho1-2*, *pho1;h3-1, pho1;h3-2*, and *pho1-2/pho1;h3-1* mutant plants as well as transgenic *pho1;h3-1* mutant plants expressing a PHO1;H3::GFP fusion (PHO1;H3c) were grown vertically on agar-solidified media containing 1mM Pi and 15 μM Zn (+Pi,+Zn), 15 μM Zn and no Pi (–Pi,+Zn), or 1mM Pi and no Zn (+Pi,–Zn). Pi concentrations were quantified in shoots of 20-d-old plants. Individual measurements were obtained from the analysis of shoots collected from a pool of at least 10 plants. Error bars correspond to standard deviation from three biological repeats. Asterisks indicate statistically significant differences compared to the +Pi +Zn treatment within each genotype (*P* <0.05). Hashes indicate statistically significant differences between wild type and mutants under –Zn condition.

PHO1;H3 restricts root-to-shoot Pi transfer requiring PHO1 function for Pi homeostasis under Zn deficiency. So far *PHO1* homologues have not been tested for their functionality in response to different nutritional stresses other than Pi deprivation. Considering the results presented above, this work checked whether *PHO1;H3* could play a role in Pi homeostasis under Zn deficiency. Two independent *pho1;h3* knockout lines have been selected from the T-DNA mutant collection. Expression of *PHO1;H3* was shown to be abolished in both lines (data not shown). In order to analyse the role of *PHO1;H3* in transport of Pi within the plant, this work measured Pi content of shoots and roots in wild-type and *pho1;h3* mutant plants (Fig. 8). Under full nutrient conditions, wild-type and *pho1;h3* mutant plants showed similar Pi contents in the shoot. While Zn-deficient wild-type plants accumulated 18% more Pi compared to Zn-sufficient conditions, the Zn-deficient *pho1;h3* mutant lines accumulated 30% more Pi content compared to Zn-sufficient conditions. This hyper-Pi accumulation phenotype of the *pho1;h3* mutant was abolished by the expression of the *PHO1;H3-GFP* fusion construct under the control of its native promoter (Fig. 8). Altogether, these results indicate that *PHO1;H3* restricts the transport of Pi from the roots to the shoot in response to a Zn-deficiency.

**Fig. 9.**
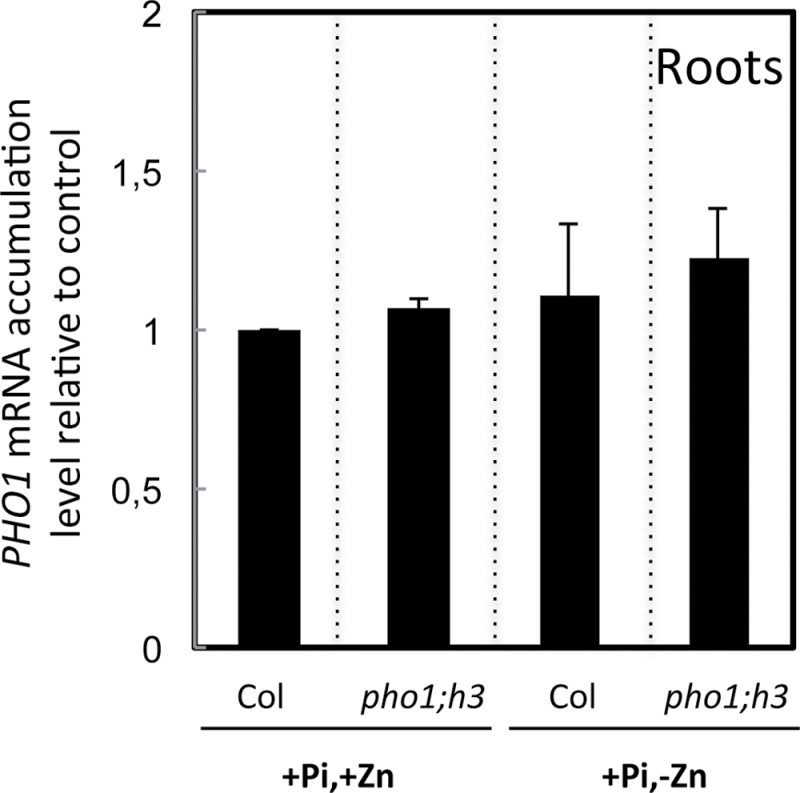
mRNA accumulation of the *PHO1* gene in response to the availability of Zn in wild-type and *pho1;h3* mutant plants. Plants were grown vertically on agar-solidified media containing 1mM Pi and 15 μM Zn (+Pi,+Zn), 15 μM Zn and no Pi (–Pi,+Zn), or 1mM Pi and no Zn (+Pi,–Zn). Roots of 20-d-old plants were separately harvested and mRNA accumulation was quantified by quantitative reverse-transcription PCR. mRNA abundance was normalized to the mRNA abundance of the *UBQ10* control gene and expressed as relative values against wild-type plants grown in +Pi,+Zn medium. Individual measurements were obtained from the analysis of roots collected from a pool of at least 10 plants. Error bars correspond to standard deviation from three biological replicates.

The *PHO1* transcript accumulation level was assessed in wild-type plants and *pho1;h3* mutants grown in presence or absence of Zn. The results show that *PHO1* transcript level in either the wild type or *pho1,h3* mutant was not significantly influenced by the presence or absence of Zn in the growth media (Fig. 9). Furthermore, the *PHO1* mRNA accumulation level did not change in the *pho1;h3* mutant in comparison to wild-type plants (Fig. 9). These results show that *PHO1* is not regulated at the transcriptional level either by the availability of Zn or by the presence of *PHO1;H3*.

Considering the similarity in the localization and expression of PHO1;H3 and PHO1, PHO1;H3 can be hypothesized to be involved in the regulation of Pi loading into root xylem via an interaction with PHO1. In order to test this hypothesis, a *pho1 pho1;h3* double mutant was created. The *pho1 pho1;h3* double mutant accumulated the same levels of Pi in the shoot as the *pho1* mutant regardless of the availability of Zn in the culture medium (Fig. 8). This indicated that the contribution of PHO1;H3 to the control of the Pi shoot content in response to Zn deficiency requires PHO1.

## Discussion

To date, research in the field of Pi and Zn nutrition in plants has mainly considered each of these nutrients separately (Poirier and Bucher, 2002; Sinclair and Krämer, 2012). However, Pi and Zn transport and homeostasis are highly coregulated processes. The existence of complex interactions linking the regulations of the homeostasis of the two nutrients has been recognized (Marschner and Cakmak, 1986; Webb and Loneragan, 1988, 1990; Norvell and Welch, 1993; Huang *et al.*, 2000). In both dicotyledons and monocotyledons, Zn deficiency has been associated with overaccumulation of Pi in shoots. Despite Zn and Pi being of fundamental importance for plant growth, the biological significance and the genetic components involved in this Zn–Pi interaction remains undetermined. Although it is expected that a certain level of coordination and crosstalk must exist between pathways involved in Pi and Zn transport and signalling in plants, key genes acting in this coordination still remain to be identified. As shown here, the model plant *A. thaliana* also overaccumulates Pi in shoots under Zn deprivation. Interestingly, the *Arabidopsis* Pi-signalling transcription factor *PHR1* appears to be necessary for this control, thus providing evidence in favour of a genetic basis for the interconnection between Zn and Pi homeostasis in *Arabidopsis*. *PHR1* is a master gene controlling the expression of numerous genes under Pi-deficient conditions, including the so-called ‘PHR1–miR399–PHO2’ Pi-starvation-signalling pathway (Rubio *et al.*, 2001; Franco-Zorrilla et al., 2004, 2007; Aung *et al.*, 2006; Bari *et al.*, 2006; Chiou *et al.*, 2006; Lin *et al.*, 2008; Pant *et al.*, 2008; Bustos *et al.*, 2010). However, this signalling pathway and some of its target genes did not appear to be active under Zn limitation either in shoots or in roots (Figs. 1 and 2; Jain *et al.*, 2013). In particular, mutation in *PHO2* did not abolish the Pi overaccumulation response to Zn deprivation. Altogether, these results revealed that *PHR1* plays a role in Zn–Pi interaction but ruled out the involvement of the already known PHR1–miR399–PHO2 Pi-starvation signalling cascade in this process. In this context, it is worth noting that besides being involved in the regulation of Pi homeostasis, *PHR1* is directly or indirectly involved in the control of many other metabolic processes, such as sulphate transport (Rouached *et al.*, 2011*a*), ROS scavenging and detoxification, and light reactions of photosynthesis and photorespiration (Bustos *et al.*, 2010). Interestingly, Bournier *et al.* (2013) very recently demonstrated that *PHR1* regulates the expression of *Arabidopsis FERRITIN 1* and is thus is a key element in the coordination of iron and Pi homeostasis. In this context, PHR1 can be hypothesized as a central player coordinating the interaction between Pi and metals in plants. The next task will be to discover the new signalling pathways involving *PHR1* in the coordination of the Zn–Pi homeostasis to develop a comprehensive understanding of this process.

The identification of key genes involved in the coregulation of micro- and macronutrient homeostasis has become a major focal interest. In this work, in addition to *PHR1*, *PHO1* appeared as another player for the Zn–Pi interaction. *PHO1* was necessary for the increase in Pi accumulation in the shoot under Zn deficiency. Considering that PHO1 is involved in Pi loading in the xylem, the results suggest that PHO1 could be a target in the Zn–Pi interconnection signalling pathway as detailed below. The other potential targets of the Zn–Pi signalling interaction that were examined were the high-affinity Pi transporters. In barley, Zn deprivation applied to plants grown with an adequate supply of Pi was shown to induce the expression in roots of genes encoding high-affinity Pi transporters, which are usually overexpressed only in response to Pi deficiency (Huang *et al.*, 2000). Interestingly, in *A. thaliana*, no significant difference in the transcript accumulation of the *PHT1* high-affinity Pi transporters in roots could be observed in response to Zn deprivation in these experimental conditions (Fig. 3). This finding was in agreement with transcriptomic data showing that the expression levels of the *PHT1* genes were not affected by the application of Zn deprivation (Van de Mortel *et al.*, 2006). Collectively, these results indicate that the regulation of Pi acquisition from the soil solution may differ between plants species in response to Zn deficiency. It is, however, interesting to point that unexpectedly the transcript abundance of *PHT1;1* is upregulated in the shoots of *Arabidopsis* Zn-deficient plants. The hypothesized primary function of PHT1;1 is the uptake of Pi from the soil and apoplastic space, particularly in roots (Nussaume *et al.*, 2011). Its precise physiological role in shoots and regulation by Zn status requires further investigation. More studies on regulation of PHT1 protein accumulation and Pi transport activity are required to explain contribution of these Pi transporters under Zn deficiency.

This work identified *PHO1;H3* as an important player in the crosstalk between Zn deficiency signalling and the regulation of Pi homeostasis in *Arabidopsis*. Indeed, *PHO1;H3* was significantly induced by Zn deficiency (Fig. 4). Furthermore, the *pho1;h3* mutant accumulated similar levels of Pi as the wild type in the control condition, but accumulated significantly more Pi than the wild type when grown in Zn-free medium (Fig. 8). This results is intriguing, since the similarity in the expression pattern and subcellular localization of PHO1 and PHO1;H3 (Figs. 6 and 7) could be interpreted as an indication that both genes play a similar role in Pi transport from root to shoot. However, previous studies have shown that expression of *PHO1;H3* under the control of the *PHO1* promoter failed to complement the *pho1* mutant (Stefanovic *et al.*, 2007). An attractive hypothesis to explain the results of this work would be that *PHO1;H3* negatively regulates PHO1 activity in Pi transport to the shoot under Zn deficiency. This hypothesis is supported by the observation that PHO1 appears to be necessary for the overaccumulation of Pi in the shoot in response to Zn deprivation and that the *pho1 pho1;h3* double mutant displays an equivalent Pi content in the shoot as the *pho1* mutant regardless of the availability of Zn in the culture medium (Fig. 8). It is tempting to speculate that PHO1;H3 and PHO1 may directly interact to regulate the transfer of Pi to the shoot in response to Zn limitation. The modulation of the activity of PHO1 as a result of a protein–protein interaction has already been reported (Liu *et al.*, 2012). This process involves PHO2, an E2-conjugase, which was shown to be a negative regulator of PHO1 activity via its degradation. PHO1 and PHO2 physically interact to regulate – and restrict – the transfer of Pi to the shoot depending on the Pi status of the plant. Under Pi deficiency, the abundance of the PHO2 protein is reduced, which leads to an increase in the accumulation of the PHO1 protein and consequently enhances the capacity of the plant to transfer Pi to the shoot. Plants have the ability to adjust their Pi absorption capacity to maintain Pi concentration within physiological limits in their tissues. In this respect, PHO2 contributes to limit Pi overaccumulation and the *pho2* mutant is characterized by Pi overaccumulation in shoots, leading to Pi toxicity (Delhaize and Randall, 1995; Dong *et al.*, 1998; Aung *et al.*, 2006). Interestingly, the present work suggests that modulation of PHO1;H3 may be another mechanism limiting excessive Pi accumulation. It will be necessary to further investigate whether PHO1;H3 interacts directly with PHO1 and as a result can modulate its activity in response to Zn availability. In *Arabidopsis*, it has already been shown for sulphate (Rouached *et al.*, 2008), potassium (Honsbein *et al.*, 2009), and water (Fetter *et al.*, 2004; Zelazny *et al.*, 2007) that the coexpression of two different members of a multigenic family of transporters can interact with each other to change their subcellular location or activity, thus regulating the transport of the corresponding nutrient. Such mechanisms are thought to fine-tune plant metabolism and growth under different environmental conditions and could also occur for Pi nutrition.

## References

[CIT0001] Alonso JM Stepanova AN Leisse TJ, 2003 Genome-wide insertional mutagenesis of *Arabidopsis thaliana* . Science 301, 653–657 12893945 10.1126/science.1086391

[CIT0002] Ames BN 1966 Assay of inorganic phosphate, total phosphate and phosphatases. Methods in Enzymology 8, 115–118

[CIT0003] Arpat AB Magliano P Wege S Rouached H Stefanovic A Poirier Y 2012 Functional expression of PHO1 to the Golgi and trans-Golgi network and its role in export of inorganic phosphate. The Plant Journal 71, 479–491 22449068 10.1111/j.1365-313X.2012.05004.x

[CIT0004] Aung K Lin S Wu C Huang Y Su C Chiou T 2006 pho2, a phosphate overaccumulator, is caused by a nonsense mutation in a microRNA399 target gene. Plant Physiology 141, 1000–1011 16679417 10.1104/pp.106.078063 PMC1489903

[CIT0005] Batistic O Waadt R Steinhorst L Held K Kudla J 2010 CBL-mediated targeting of CIPKs facilitates the decoding of calcium signals emanating from distinct cellular stores. The Plant Journal 61, 211–222 19832944 10.1111/j.1365-313X.2009.04045.x

[CIT0006] Bari R Datt Pant B Stitt M Scheible WR 2006 PHO2, microRNA399, and PHR1 define a phosphate-signaling pathway in plants. Plant Physiology 141, 988–999 16679424 10.1104/pp.106.079707 PMC1489890

[CIT0007] Baxter I Tchieu J Sussman MR Boutry M Palmgren MG Gribskov M Harper JF Axelsen KB 2003 Genomic comparison of P-type ATPase ion pumps in *Arabidopsis* and rice. Plant Physiology 132, 618–628 12805592 10.1104/pp.103.021923 PMC167002

[CIT0008] Bournier M Tissot N Mari S Boucherez J Lacombe E Briat JF Gaymard F 2013 *Arabidopsis* FERRITIN 1 (*AtFer1*) gene regulation by the PHOSPHATE STARVATION RESPONSE 1 (AtPHR1) transcription factor reveals a direct molecular link between iron and phosphate homeostasis. Journal of Biological Chemistry 288, 22670–22680 23788639 10.1074/jbc.M113.482281 PMC3829352

[CIT0009] Bustos R Castrillo G Linhares F Puga MI Rubio V Pérez-Pérez J Solano R Leyva A PazAres J 2010 A central regulatory system largely controls transcriptional activation and repression responses to phosphate starvation in *Arabidopsis* . PLOS Genetics 6, e1001102 20838596 10.1371/journal.pgen.1001102 PMC2936532

[CIT0010] Cakmak I Marschner H 1986 Mechanism of phosphorus-induced zinc deficiency in cotton. I. Zinc deficiency-enhanced uptake rate of phosphorus. Physiologia Plantarum 68, 483–490

[CIT0011] Chiou TJ Aung K Lin SI Wu CC Chiang SF Su CL 2006 Regulation of phosphate homeostasis by microRNA in *Arabidopsis* . The Plant Cell 18, 412–421 16387831 10.1105/tpc.105.038943 PMC1356548

[CIT0012] Clough SJ Bent AF 1998 Floral dip: a simplified method for *Agrobacterium*-mediated transformation of *Arabidopsis thaliana* . The Plant Journal 6, 735–743 10069079 10.1046/j.1365-313x.1998.00343.x

[CIT0013] Curie C Cassin G Couch D Divol F Higuchi K Le Jean M Misson J Schikora A Czernic P Mari S 2009 Metal movement within the plant: contribution of nicotianamine and yellow stripe 1-like transporters. Annals of Botany 103, 1–11 18977764 10.1093/aob/mcn207 PMC2707284

[CIT0014] Curtis MD Grossniklaus U 2003 A gateway cloning vectors set for high-throughput functional analysis of genes *in planta* . Plant Physiology 133, 462–469 14555774 10.1104/pp.103.027979 PMC523872

[CIT0015] Delhaize E Randall PJ 1995 Characterization of a phosphate-accumulator mutant of *Arabidopsis thaliana* . Plant Physiology 107, 207–213 12228355 10.1104/pp.107.1.207 PMC161187

[CIT0016] Doerner P 2008 Phosphate starvation signaling: a threesome controls systemic P(i) homeostasis. Current Opinion in Plant Biology 11, 536–540 18614391 10.1016/j.pbi.2008.05.006

[CIT0017] Dong B Rengel Z Delhaize E 1998 Uptake and translocation of phosphate by pho2 mutant and wild-type seedlings of *Arabidopsis thaliana* . Planta 205, 251–256 9637070 10.1007/s004250050318

[CIT0018] Fetter K Van Wilder V Moshelion M Chaumont F 2004 Interactions between plasma membrane aquaporins modulate their water channel activity. The Plant Cell 16, 215–228 14671024 10.1105/tpc.017194 PMC301406

[CIT0019] Franco-Zorrilla J Gonzalez E Bustos R Linhares F Leyva A Paz-Ares J 2004 The transcriptional control of plant responses to phosphate limitation. Journal of Experimental Botany 55, 285–293 14718495 10.1093/jxb/erh009

[CIT0020] Franco-Zorrilla JM Valli A Todesco M Mateos I Puga MI Rubio-Somoza I Leyva A Weigel D Garcia JA Paz-Ares J 2007 Target mimicry provides a new mechanism for regulation of microRNA activity. Nature Genetics 39, 1033–1037 17643101 10.1038/ng2079

[CIT0021] Fujii H Chiou TJ Lin SI Aung K Zhu JK 2005 A miRNA involved in phosphate-starvation response in *Arabidopsis* . Current Biology 15, 2038–2043 16303564 10.1016/j.cub.2005.10.016

[CIT0022] Grotz N Fox T Connolly E Park W Guerinot ML Eide D 1998 Identification of a family of zinc transporter genes from *Arabidopsis* that respond to zinc deficiency. Proceedings of the National Academy of Sciences, USA 95, 7220–7224 10.1073/pnas.95.12.7220 PMC22785 9618566

[CIT0023] Guerinot ML 2000 The ZIP family of metal transporters. Biochimica et Biophysica Acta 1465, 190–198 10748254 10.1016/s0005-2736(00)00138-3

[CIT0024] Hall JL Williams LE 2003 Transition metal transporters in plants. Journal of Experimental Botany 54, 2601–2613 14585824 10.1093/jxb/erg303

[CIT0025] Hamburger D Rezzonico E MacDonald-Comber Petetot J Somerville C Poirier Y 2002 Identification and characterization of the *Arabidopsis* PHO1 gene involved in phosphate loading to the xylem. The Plant Cell 14, 889–902 11971143 10.1105/tpc.000745 PMC150690

[CIT0026] Honsbein A Sokolovski S Grefen C Campanoni P Pratelli R Paneque M Chen Z Johansson I Blatt MR 2009 A tripartite SNARE-K+ channel complex mediates in channel-dependent K+ nutrition in *Arabidopsis* . The Plant Cell 21, 2859–2877 19794113 10.1105/tpc.109.066118 PMC2768940

[CIT0027] Hsieh LC Lin SI Shih AC Chen JW Lin WY Tseng CY Li WH Chiou TJ 2009 Uncovering small RNA-mediated responses to phosphate deficiency in *Arabidopsis* by deep sequencing. Plant Physiology 151, 2120–2132 19854858 10.1104/pp.109.147280 PMC2785986

[CIT0028] Huang C Barker SJ Langridge P Smith FW Graham RD 2000 Zinc deficiency up-regulates expression of high-affinity phosphate transporter genes in both phosphate-sufficient and -deficient barley roots. Plant Physiology 124, 415–422 10982454 10.1104/pp.124.1.415 PMC59154

[CIT0029] Hussain D Haydon MJ Wang Y Wong E Sherson SM Young J Camakaris J Harper JF Cobbett CS 2004 P-type ATPase heavy metal transporters with roles in essential zinc homeostasis in *Arabidopsis* . The Plant Cell 16, 1327–1339 15100400 10.1105/tpc.020487 PMC423219

[CIT0030] Jain A Sinilal B Dhandapani G Meagher RB Sahi SV 2013 Effects of deficiency and excess of zinc on morphophysiological traits and spatiotemporal regulation of zinc-responsive genes reveal incidence of cross talk between micro- and macronutrients. Environmental Science and Technology 47, 5327–5335 23590825 10.1021/es400113y

[CIT0031] Kang X Ni M 2006 *Arabidopsis* SHORT HYPOCOTYL UNDER BLUE1 contains SPX and EXS domains and acts in cryptochrome signaling. The Plant Cell 18, 921–934 16500988 10.1105/tpc.105.037879 PMC1425848

[CIT0032] Lagarde D Basset M Lepetit M Conejero G Gaymard F Astruc S Grignon C 1996 Tissue-specific expression of *Arabidopsis* AKT1 gene is consistent with a role in K+ nutrition. The Plant Journal 9, 195–203 8820606 10.1046/j.1365-313x.1996.09020195.x

[CIT0033] Leach BS Collawn JF Fish WW 1980 Behavior of glycopolypeptides with empirical molecular weight estimation methods. Biochemistry 19, 5734–6747 7459341 10.1021/bi00566a011

[CIT0034] Lin SI Chiang SF Lin WY Chen JW Tseng CY Wu PC Chiou TJ 2008 Regulatory network of microRNA399 and PHO2 by systemic signaling. Plant Physiology 147, 732–746 18390805 10.1104/pp.108.116269 PMC2409027

[CIT0035] Liu TY Huang TK Tseng CY Lai YS Lin SI Lin WY Chen JW Chiou TJ 2012 PHO2-dependent degradation of PHO1 modulates phosphate homeostasis in *Arabidopsis* . The Plant Cell 24, 2168–2183 22634761 10.1105/tpc.112.096636 PMC3442594

[CIT0036] Livak KJ Schmittgen TD 2001 Analysis of relative gene expression data using real-time quantitative PCR and the 2(-Delta Delta C(T)) method. Methods 25, 402–408 11846609 10.1006/meth.2001.1262

[CIT0037] Loneragan JK Grunes DL Welch RM Aduayi EA Tengah A Lazar VA Cary EE 1982 Phosphorus accumulation and toxicity in leaves in relation to zinc supply. Soil Science Society of America Journal 46, 345–352

[CIT0038] Loneragan JF Webb MJ 1993 Interactions between zinc and other nutrients affecting the growth of plants. In: Robson AD, editor, Zinc in soils and plants . Dordrecht, The Netherlands: Kluwer Academic Publishers, pp 119–134

[CIT0039] Marschner H Cakmak I 1986 Mechanism of phosphorus-induced zinc deficiency in cotton. II. Evidence for impaired shoot control of phosphorus uptake and translocation under zinc deficiency. Physiologia Plantarum 68, 491–496

[CIT0040] Martín AC del Pozo JC Iglesias J Rubio V Solano R De La Peña A Leyva A Paz-Ares J 2000 Influence of cytokinins on the expression of phosphate starvation responsive genes in *Arabidopsis* . The Plant Journal 24, 1–11 11123795 10.1046/j.1365-313x.2000.00893.x

[CIT0041] Mäser P Thomine S Schroeder JI, 2001 Phylogenetic relationships within cation transporter families of *Arabidopsis* . Plant Physiology 126, 1646–1667 11500563 10.1104/pp.126.4.1646 PMC117164

[CIT0042] Muchhal US Raghothama KG 1999 Transcriptional regulation of plant phosphate transporters. Proceedings of the National Academy of Sciences, USA 96, 5868–5872 10.1073/pnas.96.10.5868 PMC21952 10318976

[CIT0043] Nebenfuhr A Gallagher LA Dunahay TG Frohlick JA Mazurkiewicz AM Meehl JB Staehelin LA 1999 Stop-and-go movements of plant Golgi stacks are mediated by the acto-myosin system. Plant Physiology 121, 1127–1141 10594100 10.1104/pp.121.4.1127 PMC59480

[CIT0044] Nelson BK Cai X Nebenfuehr A 2007 A multicolored set of *in vivo* organelle markers for co-localization studies in *Arabidopsis* and other plants. The Plant Journal 51, 1126–1136 17666025 10.1111/j.1365-313X.2007.03212.x

[CIT0045] Norvell WA Welch RM 1993 Growth and nutrient uptake by barley (*Hordeum vulgare* L. cv Herta): studies using an N-(2-hydroxyethyl) ethylenedinitrilotriacetic acid-buffered nutrient solution technique: I. Zinc ion requirements. Plant Physiology 101, 619–625 12231717 10.1104/pp.101.2.619 PMC160611

[CIT0046] Nussaume L Kanno S Javot H Marin E Pochon N Ayadi A Nakanishi TM Thibaud MC 2011 Phosphate import in plants: focus on the PHT1 transporters. Frontiers in Plant Science 2, 83 22645553 10.3389/fpls.2011.00083 PMC3355772

[CIT0047] Palmer CM Guerinot ML 2009 Facing the challenges of Cu, Fe and Zn homeostasis in plants. Nature Chemical Biology 5, 333–340 10.1038/nchembio.166 PMC3085079 19377460

[CIT0048] Pant BD Buhtz A Kehr J Scheible WR 2008 MicroRNA399 is a long-distance signal for the regulation of plant phosphate homeostasis. The Plant Journal 53, 731–738 17988220 10.1111/j.1365-313X.2007.03363.x PMC2268993

[CIT0049] Pant BD Musialak-Lange M Nuc P May P Buhtz A Kehr J Walther D Scheible WR 2009 Identification of nutrient-responsive *Arabidopsis* and rapeseed microRNAs by comprehensive real-time polymerase chain reaction profiling and small RNA sequencing. Plant Physiology 150, 1541–1555 19465578 10.1104/pp.109.139139 PMC2705054

[CIT0050] Perea-García A Garcia-Molina A Andrés-Colás N Vera-Sirera F Pérez-Amador MA Puig S Peñarrubia L 2013 *Arabidopsis* copper transport protein COPT2 participates in the cross talk between iron deficiency responses and low-phosphate signaling. Plant Physiology 162, 180–194 23487432 10.1104/pp.112.212407 PMC3641201

[CIT0051] Poirier Y Bucher M 2002 Phosphate transport and homeostasis in *Arabidopsis* . The Arabidopsis Book 1, e0024 22303200 10.1199/tab.0024 PMC3243343

[CIT0052] Poirier Y Thoma S Somerville C Schiefelbein J 1991 Mutant of *Arabidopsis* deficient in xylem loading of phosphate. Plant Physiology 97, 1087–1093 16668493 10.1104/pp.97.3.1087 PMC1081126

[CIT0053] Ribot C Wang Y Poirier Y 2008a Expression analyses of three members of the AtPHO1 family reveal differential interactions between signaling pathways involved in phosphate deficiency and the responses to auxin, cytokinin, and abscisic acid. Planta 227, 1025–1036 18094993 10.1007/s00425-007-0677-x

[CIT0054] Ribot C Zimmerli C Farmer EE Reymond P Poirier Y 2008b Induction of the *Arabidopsis* PHO1;H10 gene by 12-oxo-phytodienoic acid but not jasmonic acid via a CORONATINE INSENSITIVE1-dependent pathway. Plant Physiology 147, 696–706 18434606 10.1104/pp.108.119321 PMC2409032

[CIT0055] Robson AD Pitman MG 1983 Interactions between nutrients in higher plants. In: Lauchli A Bieleski RL, editors, Encyclopaedia of plant physiology , vol. 15A New series. Berlin and New York: Springer-Verlag, 287–312

[CIT0056] Rouached H Arpat AB Poirier Y 2010 Regulation of phosphate starvation responses in plants: signaling players and cross-talks. Molecular Plant 3, 288–299 20142416 10.1093/mp/ssp120

[CIT0057] Rouached H Secco D Arpat AB Poirier Y 2011a The transcription factor PHR1 plays a key role in the regulation of sulfate shoot-to-root flux upon phosphate starvation in *Arabidopsis* . BMC Plant Biology 24, 19 21261953 10.1186/1471-2229-11-19 PMC3036608

[CIT0058] Rouached H Stefanovic A Secco D Bulak Arpat A Gout E Bligny R Poirier Y 2011b Uncoupling phosphate deficiency from its major effects on growth and transcriptome via PHO1 expression in *Arabidopsis* . The Plant Journal 65, 557–570 21288266 10.1111/j.1365-313X.2010.04442.x

[CIT0059] Rouached H Wirtz M Alary R Hell R Arpat AB Davidian JC Fourcroy P Berthomieu P 2008 Differential regulation of the expression of two high-affinity sulfate transporters, SULTR1.1 and SULTR1.2, in *Arabidopsis* . Plant Physiology 147, 897–911 18400935 10.1104/pp.108.118612 PMC2409035

[CIT0060] Rubio V Linhares F Solano R Martín AC Iglesias J Leyva A Paz-Ares J 2001 A conserved MYB transcription factor involved in phosphate starvation signaling both in vascular plants and in unicellular algae. Genes and Development 15, 2122–2133 11511543 10.1101/gad.204401 PMC312755

[CIT0061] Safaya NM Gupta AP 1979 Differential susceptibility of corn cultivars to zinc deficiency. Agronomy Journal 71, 132–136

[CIT0062] Sanderfoot AA Kovaleva V Bassham DC Raikhel NV 2001 Interactions between syntaxins identify at least five SNARE complexes within the golgi/prevacuolar system of the *Arabidopsis* cell. Molecular Biology of the Cell 12, 3733–3743 11739776 10.1091/mbc.12.12.3733 PMC60751

[CIT0063] Shin H Shin HS Chen R Harrison MJ 2006 Loss of At4 function impacts phosphate distribution between the roots and the shoots during phosphate starvation. The Plant Journal 45, 712–726 16460506 10.1111/j.1365-313X.2005.02629.x

[CIT0064] Sinclair SA Krämer U 2012 The zinc homeostasis network of land plants. Biochimica et Biophysica Acta 1823, 1553–1567 22626733 10.1016/j.bbamcr.2012.05.016

[CIT0065] Singh JP Karamanos RE Stewart JWB 1988 The mechanism of phosphorus-induced zinc deficiency in bean (*Phaseolus vulgaris* L.). Canadian Journal of Soil Science 68, 345–358

[CIT0066] Smith FW Rae AL Hawkesford MJ 2000 Molecular mechanisms of phosphate and sulphate transport in plants. Biochimica et Biophysica Acta 1465, 236–245 10748257 10.1016/s0005-2736(00)00141-3

[CIT0067] Sridhar VV Kapoor A Zhang KL Zhu JJ Zhou T Hasegawa PM Bressan RA Zhu JK 2007 Control of DNA methylation and heterochromatic silencing by histone H2B deubiquitination. Nature 447, 735–738 17554311 10.1038/nature05864

[CIT0068] Stefanovic A Ribot C Rouached H Wang Y Chong J Belbahri L Delessert S Poirier Y 2007 Members of the PHO1 gene family show limited functional redundancy in phosphate transfer to the shoot, and are regulated by phosphate deficiency via distinct pathways. The Plant Journal 50, 982–994 17461783 10.1111/j.1365-313X.2007.03108.x

[CIT0069] Van de Mortel JE Almar Villanueva L Schat H Kwekkeboom J Coughlan S Moerland PD Ver Loren van Themaat E Koornneef M Aarts MG 2006 Large expression differences in genes for iron and zinc homeostasis, stress response, and lignin biosynthesis distinguish roots of *Arabidopsis thaliana* and the related metal hyperaccumulator *Thlaspi caerulescens* . Plant Physiology 142, 1127–1147 16998091 10.1104/pp.106.082073 PMC1630723

[CIT0070] Verma TS Minhas RS 1987 Zinc and phosphorus interaction in a wheat–maize cropping system. Fertilizer Research 13, 77–86

[CIT0071] Wang Y Ribot C Rezzonico E Poirier Y 2004 Structure and expression profile of the *Arabidopsis* PHO1 gene family indicates a broad role in inorganic phosphate homeostasis. Plant Physiology 135, 400–411 15122012 10.1104/pp.103.037945 PMC429393

[CIT0072] Webb WJ Loneragan JF 1988 Effect of zinc deficiency on growth, phosphorus concentration, and phosphorus toxicity of wheat plants. Soil Science Society of America Journal 52, 1676–1680

[CIT0073] Webb WJ Loneragan JF 1990 Zinc translocation to wheat roots and its implications for a phosphorus/zinc interaction in wheat plants. Journal of Plant Nutrition 13, 1499–1512

[CIT0074] Weber M Harada E Vess C Roepenack-Lahaye E Clemens S 2004 Comparative microarray analysis of *Arabidopsis thaliana* and *Arabidopsis halleri* roots identifies nicotianamine synthase, a ZIP transporter and other genes as potential metal hyperaccumulation factors. The Plant Journal 37, 269–281 14690510 10.1046/j.1365-313x.2003.01960.x

[CIT0075] Zelazny E Borst JW Muylaert M Batoko H Hemminga MA Chaumont F 2007 FRET imaging in living maize cells reveals that plasma membrane aquaporins interact to regulate their subcellular localization. Proceedings of the National Academy of Sciences, USA 104, 12359–12364 10.1073/pnas.0701180104 PMC1941474 17636130

[CIT0076] Zhou Y Ni M 2009 SHB1 plays dual roles in photoperiodic and autonomous flowering. Developmental Biology 331, 50–57 19406114 10.1016/j.ydbio.2009.04.023

[CIT0077] Zhou Y Zhang X Kang X Zhao X Zhang X Ni M 2009 SHORT HYPOCOTYL UNDER BLUE1 associates with MINISEED3 and HAIKU2 promoters *in vivo* to regulate *Arabidopsis* seed development. The Plant Cell 21, 106–117 19141706 10.1105/tpc.108.064972 PMC2648090

[CIT0078] Zhu YG Smith SE Smith FA 2001 Plant growth and cation composition of two cultivars of spring wheat (*Triticum aestivum* L.) differing in P uptake efficiency. Journal of Experimental Botany 52, 1277–1282 11432946

